# A Smart Data Hub to integrate subsurface properties into model-based decision-support tools

**DOI:** 10.1038/s41597-026-07580-x

**Published:** 2026-06-23

**Authors:** Qian Chen, Nino Menzel, Marc S. Boxberg, Julia Kowalski

**Affiliations:** 1 Methods for Model-based Development in Computational Engineering, RWTH Aachen University, Aachen, Germany; 2 Geophysical Imaging and Monitoring, RWTH Aachen University, Aachen, Germany

## Abstract

The selection of suitable nuclear waste disposal sites is a long and challenging process, involving multiple technical and environmental assessments. For data-integrated simulation models to function as credible decision-support tools in this sensitive context, their data sources must meet rigorous transparency and reproducibility standards. This paper introduces a Smart Data Hub that provides the reproducible data foundation required to address the limits found in existing research, which rarely features both a dataset containing uncertainty information and an effective way of application for specific use cases. The Smart Data Hub is an integrated solution consisting of two main components: a dataset compiled from 50 literature sources covering geological information, structural data, and rock properties for potential repository sites in Germany, and a functional module supporting effective assembly of data compilations for a given geological structure. This approach provides reliable, reproducible data compilation for model-based decision support in the site selection process by providing transparent, uncertainty-informed data coupled with intelligent assembly capabilities.

## Background & Summary

After decades of using nuclear power commercially, not much of it remains as Germany is transitioning into using green energy^1^. One aspect that has remained and will remain so for thousands or even millions of years is high-level radioactive waste. Currently, the only solution is to dispose it underground for the foreseeable future until it becomes harmless. Although the process is defined in the Standortauswahlgesetz (StandAG), or Site Selection Act^2^, finding the right location is difficult. The right place entails successfully integrating multiple geological factors, including very low rock permeability, sufficient thickness and depth, sufficient area for repository construction, and demonstrated viability over long-term timescales as specified in the Endlagersicherheitsanforderungsverordnung, or Final Storage Safety Ordinance^3^. Given the number of variables present, setting up testing sites to test their long-term performance is not feasible. This fact, however, moves the spotlight onto complementary simulations, which in turn are used to narrow down possible sites by providing a detailed outlook on the future potential of each individual area, e.g., simulation-based assessment of radioactive contamination through groundwater flow. These simulations enable an assessment of site suitability prior to committing any noteworthy amount of monetary or resource investments, making them a key component of the data-driven decision-making process. Naturally, the outcome of such a simulation is only as good as its input—the data provided.

This data has to enable effective assembly into compilations that match specific geological structures and geographic areas, which demands an effective tagging system capable of filtering and organizing information while preserving data integrity throughout the process. The task becomes more complex when taking into account variations in rock properties depending on location and geological age, creating uncertainties that directly influence how nuclear waste interacts with its environment over extended periods of time. Simply acknowledging the existence of these uncertainties is not sufficient. Instead, they must be quantified and integrated into analytical frameworks^4^. Moreover, given the long-term consequences of repository site selection, every simulation must abide by principles of transparency and reproducibility, enabling independent verification by third parties. To sum up, four focal points arise: First, data collection from existing sources or dedicated campaigns must be comprehensive and extend beyond a single source for each needed parameter, even if this leads to redundant or even overdetermined data to describe a certain rock type. A direct consequence of this is that there might be inconsistencies and conflicts in the provided information, say, various permeability values per rock type. The second focal point, hence, is to provide strategies to address this challenge, either by considering data configurations associated with a certain model setup based on a manually selected subset of the data or by designing intelligent compilation strategies. The benefit of a comprehensive data point is that it contains information on variability within a parameter of interest, facilitating explicit forward and backward uncertainty quantification, which constitutes our third focal point. Finally, the fourth focal point ensures that the data-integrated workflow functions as part of a model-based decision-support tool that enforces a high level of transparency and reproducibility standards. At a more general level, information management is tackled by the FAIR Guiding Principles, a framework for scientific data management across multiple domains. According to the guidelines, datasets have to meet the following four guiding principles: Findability, Accessibility, Interoperability, and Reusability, to ensure transparency, reproducibility and reusability^5^. A long-standing example of such a metadata dataset is the GFZ Data Services by the GFZ Helmholtz Centre for Geosciences, which has been running since 2004 and strictly abides by the FAIR principles. While not specific to radioactive waste disposal, it demonstrates the practical maturity of such an implementation^6^. An international example by the Nuclear Energy Agency is the Radioactive Waste Repository Metadata Management initiative^7^, launched in 2014 to harmonize metadata across 12 nuclear waste management organizations, and its successor, the Working Party on Information, Data and Knowledge Management, which expanded this work into a holistic framework for Information, Data and Knowledge Management for radioactive waste management and decommissioning^8^. At a European programme level, the European Joint Programme on Radioactive Waste Management (EURAD), running from 2019 to 2024, included multiple work packages on knowledge management with a specific goal of creating a common framework to structure knowledge^9^. This framework would then be picked up afterward by EURAD-2, which already published a formal data management plan^10^. While the above-mentioned initiatives laid the groundwork in terms of frameworks and common vocabularies, the translation into operational, site-specific datasets requires addressing practical constraints and the means to overcome them, which are the focus of this work.

When addressing practical constraints, those related to data are most significant. Because, while substantial data exists, it is characterized by significant fragmentation due to the distribution of datasets across multiple agencies, each collecting data for diverse projects. An example of how multiple agencies with overlapping research can cause inefficiencies and information scattering can be seen in the following. In the federal state of Baden-Württemberg, which hosts possible repository locations, the Landesamt für Geologie, Rohstoffe und Bergbau, or State Office for Geology, Raw Materials, and Mining, provides valuable geological maps for the state^11^. These maps, however, are artificially constrained by political borders despite the transboundary nature of geological formations. The Bundesanstalt für Geowissenschaften und Rohstoffe (BGR), or Federal Institute for Geosciences and Natural Resources, provides federal-level coordination and centralized data in some instances, as is the case with the creation of a map containing drilling locations in Germany^12^. Besides governmental institutions, important providers of geological information are research institutions. A noteworthy example is the geophysical information system FIS Geophysics by the Leipzig Institute for Applied Science^13^, serving as a dataset containing petrophysical and borehole information from Germany. Nevertheless, current data management demonstrates deficiencies regarding effective data compilation strategies and the capturing of uncertainties. This issue further transcends into research where data may be compiled, but important metainformation, including detailed descriptions, involved agencies, or location, is too often not recorded^14–17^. Consequently, researchers face numerous obstacles when considering the fragmented data landscape. The first challenge is data availability, and when it is available, it is scattered across a diverse number of institutions, raising barriers to efficient data compilation for simulations. The process gets only further complicated by a plethora of licensing policies. Moreover, current datasets lack an adequate structure to support simulations required for site evaluation in accordance with minimum repository requirements. Simultaneously, only limited uncertainty information is provided. The Smart Data Hub (SDH), in turn, cuts down on these constraints, with data contained in the SDH being published under CC BY license, enabling efficient compilation of data for specific simulations while incorporating uncertainty quantification.

**Fig. 1:**
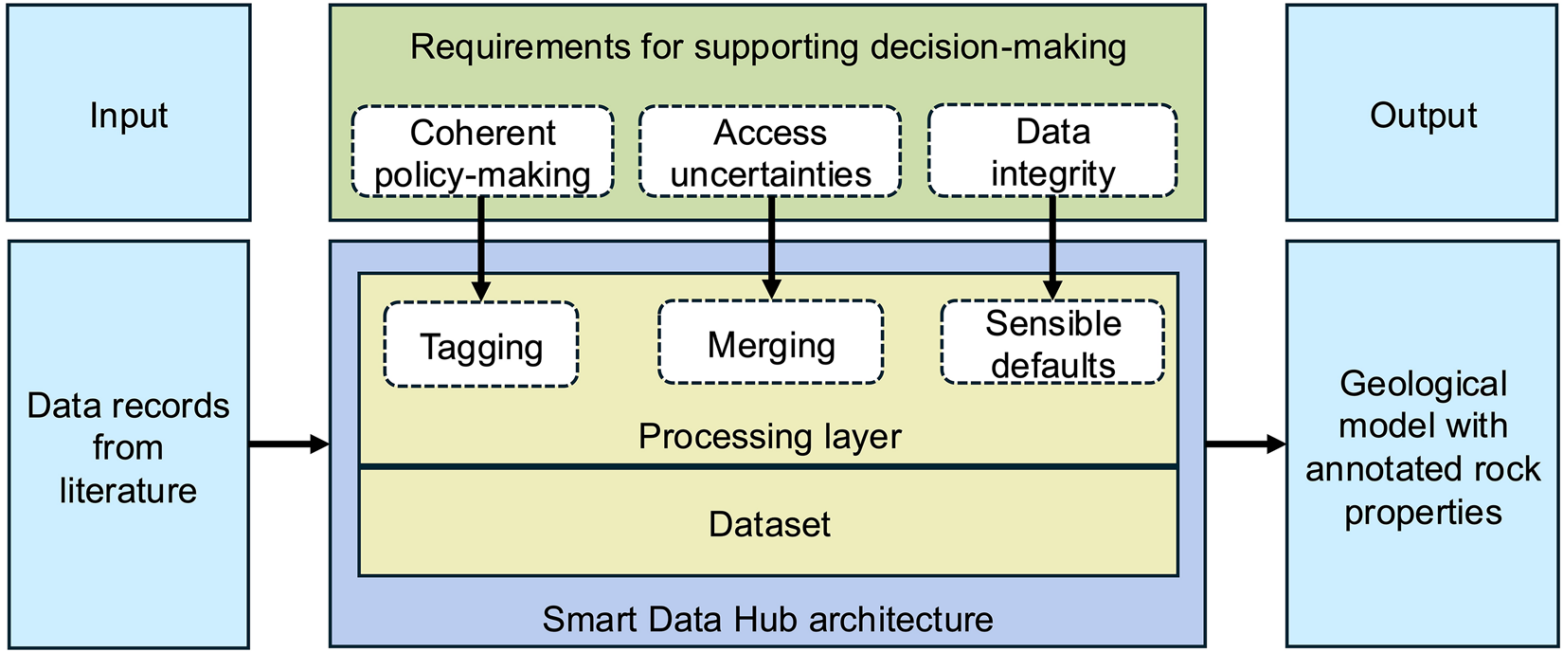
Smart Data Hub structure with formulated requirements and external points of interaction. The SDH transforms scattered geological datasets as input into tailored simulation outputs. It does so through its two-layered architecture: 1) Dataset: Compiled from literature sources, with region- and rock-type-specific petrophysical properties. 2) Processing layer: Implementation of tagging for coherent data management, merging for data integration with account for uncertainties, and sensible defaults to ensure data integrity.

Figure 1 provides an overview of the SDH architecture with its formulated requirements and external points of interaction. The overarching SDH consists of two core components: a dataset and a processing layer. The dataset was compiled from 50 different literature sources^14–63^, systematically organized by geographic subareas with their corresponding host rocks. Each regional entry contains detailed information on density, porosity, hydraulic conductivity, intrinsic permeability, specific heat capacity, thermal conductivity, P-wave velocity, S-wave velocity, and electrical resistivity, accompanied by uncertainty quantification based on rock type and age. Increasing the SDH functionality includes methods for tagging and merging of data, as well as sensible defaults. The tagging method enables users to effectively manage metadata by filtering relevant data and consolidating information for specific simulations. The merging process allows aggregation of properties from different sources while accounting for data uncertainties and gaps. Whenever data is missing, sensible defaults can fill the gap, ensuring data integrity.

While the research primarily pertains to Germany, its implications extend globally. The value of reusable data remains particularly valuable across Europe as geological formations span political borders. Prime examples are Switzerland’s Mont Terri Underground Research Laboratory and the Benken borehole, both able to provide data relevant to the Opalinus Clay area extending into southern Germany. This, in turn, means that data in our dataset serve broader European geological applications.

The SDH demonstrates inherent versatility, extending its applicability beyond nuclear waste disposal. For other fields, especially those related to transitioning towards more sustainability, such as CO2 storage, geothermal energy, and groundwater management, the SDH design proves equally valuable. This versatility greatly increases the value of the system and encourages joint development through diverse user communities.

In summary, the SDH collects and centralizes data and tags simulations according to the data involved and contained in the SDH. This systematic approach addresses limiting factors in this field, enabling efficient data compilation for specific simulations while incorporating uncertainty quantification. Increasing transparency and reproducibility are key whenever a data-integrated simulation is applied as a basis for decision-making. By leveraging the SDH, independent third parties can reproduce results from previous simulations while enjoying the benefits of a single, easily accessible data hub. As Germany continues tackling nuclear waste management challenges, the SDH provides a solid foundation for integrating data describing subsurface properties into reliable model-based decision-support tools, enabling policymakers to better account for safety and environmental considerations when deciding on a final repository location. Given the systemic approach of the SDH, its scope of application reaches far beyond Germany. Immediate beneficiaries of the SDH in this context may be countries in close proximity, e.g., those facing a similar issue and sharing similar geological features. With the input data adjustable by the end user, the output will enable a wide range of possible applications, both geographic and geological.

## Methods

This section outlines the framework created for systematic geological data compilation and integration for nuclear waste repository site assessment. This approach tackles current limitations in terms of data accessibility and fragmentation through leveraging comprehensive compilation and processing methodologies.

### Data Collection

Accurate prediction of the long-term behaviour and future risks of subsurface repository sites is essential for supporting decision-making processes for repository location and design. Physics-based simulations that explicitly account for uncertainties in geological and hydrothermal environments are employed to quantify potential radioactive contamination in groundwater^4,64,65^. Optimal experimental design identifies measurement configurations that maximize information gain from geophysical surveys, such as electrical resistivity tomography and seismic refraction^63,66^ and monitoring boreholes, subsequently reducing predictive uncertainty. To obtain the parameter values critical to those simulations, we collect nine rock properties in total: density, porosity, hydraulic conductivity, intrinsic permeability, specific heat capacity, thermal conductivity, P-wave velocity, S-wave velocity, and electrical resistivity. Table 1 describes each property in further detail.

**Table 1:** Overview of collected rock properties in the SDH.

Property	Unit	Description
Density	kg m^−3^	Bulk, dry bulk, or grain density, with bulk density being primarily collected
Porosity	—	Effective or total porosity. Effective porosity is primarily collected as interconnected pores providing active flow paths for groundwater
Hydraulic conductivity	m s^−1^	Ability of a fluid to move through the porous rock medium
Intrinsic permeability	m^2^	Quantifying the permeability of the rock medium to fluid
P-wave velocity	m s^−1^	Velocity of seismic compressional (longitudinal) body waves
S-wave velocity	m s^−1^	Velocity of seismic shear (transverse) body waves
Specific heat capacity	J kg ^−1^ K^−1^	Heat required per unit mass to increase the temperature by one unit
Thermal conductivity	W m^−1^ K ^−1^	The ability of rock to conduct heat
Electrical resistivity	Ωm	Electrical resistance of the rock medium

Hydraulic conductivity depends on both the rock and fluid properties. Therefore, during the processing of data, the hydraulic conductivity *K* will be converted into intrinsic permeability *k*, given the density of fluid *ρ*_*f*_ (kg m^−3^), acceleration of gravity *g* (m s^−2^) and dynamic viscosity *μ* (m^2^ *s*^−1^) according to the following formula:

1 $$k=\frac{K\mu }{g{\rho }_{f}}$$

Developing robust and highly efficient data-integrated simulation workflows requires systematic structural management of data. Therefore, the YAML format^67^ was chosen for storing rock properties due to its readability and processing capabilities. As a non-proprietary format, YAML offers universal compatibility across operating systems and is further enhanced by its nested mapping style, which logically organizes geological data. Additionally, it can be converted to multiple working data formats in Python, such as dictionaries and Pandas DataFrames^68^, to streamline data processing.

Data was primarily sourced from governmental reports^14–17,19,22–24,26–28,30,33,34,37–40,42–50,53–55,57^ presenting results, with a focus on potential host rocks for high-level radioactive waste, related to the site selection process. As defined in the StandAG, only three types of host rocks, namely crystalline, clay, and salt rock, are eligible for final repositories, consistent with the main host rocks considered by many other countries^69^. Thus, we focus on collecting data for these three host rock areas. Complementing sources are journal articles^25,29,35,36,41,51,52,59–63^, books^18,20,21,31,32,58^, and a dataset^56^ with CC BY license to enrich the datasets and also fill gaps wherever governmental data was insufficient. For each value taken from literature, we record meta-information of the rock type, the publishing agency, the measurement location or method, and most importantly, the original data reference. The BibTeX citation key functions in this case as an intermediate identifier for the data’s origin. All sources found during the data collection are stored in a BibTeX file inside the dataset, with DOIs or URLs (if DOIS are not available) provided to each reference. Data sources not available for public download^28,45–47,57^ were directly requested from BGR. As a first step, the source in question needs to be located within the BGR online catalogue. The catalogue provides a unique signature for each source, which can then be used to request the needed source from the BGR library via e-mail. Data is recorded in three distinct steps.

#### Step 1: Candidate Site

On the candidate site level, we record corresponding lithostratigraphic information for each rock formation in a YAML file, including geological age, lithological description and simplified lithology. The YAML file also contains paths directing to the detailed rock properties and geometric data. Table 2 details the entry fields for each site.

**Table 2:** Definition of entry fields for site-specific YAML structure. The site-specific YAML file summarizes the rock formations at the site and establishes mapping paths to the subsurface properties, including their corresponding rock formation, rock properties, and geologic information.

Field	Sub-Field	Sub-Sub-Field	Data Type	Description	Additional Note
name	—	—	string	A generic candidate site name	It follows the rule of {ISO 3166 code}_{region}_{host rock type} or omitting “region” where it is not applicable
description	—	—	string	A short description for the site	
geometry_input	source	—	string	Reference to the geological structural data’s origin	The entry here corresponds with the BibTeX citation key found in source.bib in the SDH
geometry_input	description	—	string	A short description of the data	
geometry_input	point_data_path	—	string	Path to the point data	The point data is stored in a CSV file
geometry_input	orientation_data _path	—	string	Path to the orientation data	The orientation data is stored in a CSV file
rock formation	source	—	string	Reference to the geological information’s origin	The entry here corresponds with the BibTeX citation key found in source.bib in the SDH
rock formation	geological_time	econ	string		e.g., Phanerozoic
rock formation	geological_time	era	string		e.g., Mesozoic
rock formation	geological_time	period	string		e.g., Triassic
rock formation	geological_time	epoch	string		e.g., Middle
rock formation	geological_time	age	string		e.g., Anisian
rock formation	description	—	string	A short description of the rock formation	
rock formation	simplified_lithology	—	list of strings	A list of simplified lithologies	e.g., [Mudstone, Sandstone]
rock formation	property_path	—	string	Path to the property data	
rock formation	geometry_surface _output_path	—	list of strings	A list of paths to the surface meshes	The surface meshes are stored in VTK file format

#### Step 2: Geostructural Data

**Fig. 2:**
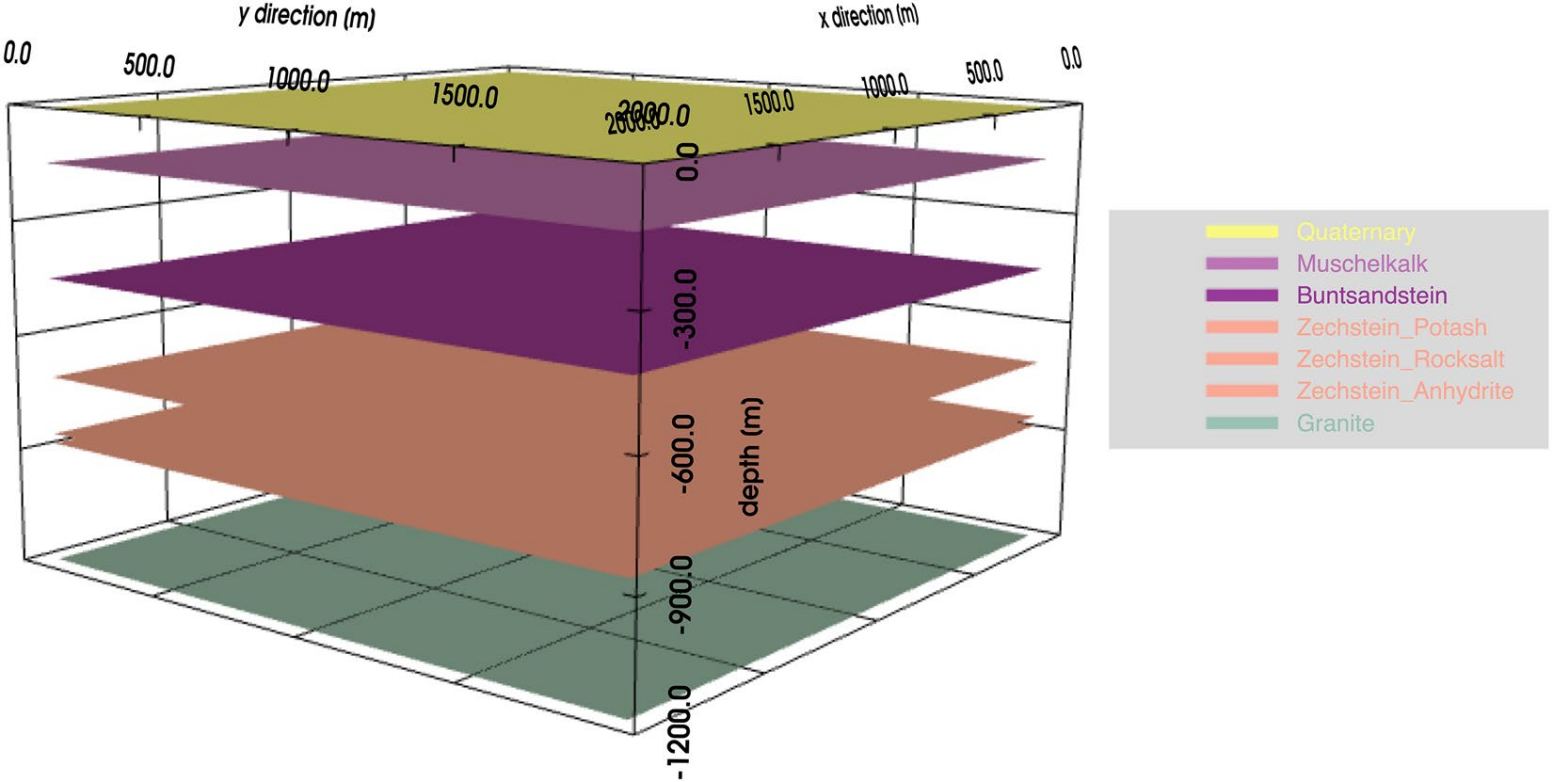
A generic geological structural model for the Crystalline host rock area. Colored interface layers indicate the bottom boundaries of the rock formations. The German terms Buntsandstein and Muschelkalk refer to the lower two stratigraphic units of the German Triassic age^99^, corresponding to the lower and parts of the middle Triassic according to the International Chronostratigraphic Chart^100^. The figure is generated using PyVista^71^.

We record geostructural information provided by the CHRISTA-II^14^, AnSichT^37,43^, and KOSINA^55^ projects for each candidate site. Geostructural information, i.e., interface and orientation points for individual lithological rock formations in a geomodel, is stored in CSV files. The 3D geological structural models were created using the GemPy^70^ and PyVista^71^ software packages based on this geostructural data. As for the defaults, universal cokriging was applied to generate the lithological interfaces^70^. These default models provided by the SDH are completely optional and can be used when the user has no model available. The 3D geological structural model provides geometric features of the subsurface, with an example in Fig. 2.

#### Step 3: Rock Properties

On the rock properties level, we record all properties defined in Table 1 for a single rock formation in a YAML file. For each data point we found in the literature, we record: the BibTex citation key indicating the data’s origin, the data value type, data value, unit in SI, uncertainty information (mean, standard deviation, or data range), and lithological information including rock type and additional tags (agency, location, and simplified lithology). Each property features a series of entries following a block mapping structure. Table 3 provides an in-depth explanation of entries within a block mapping.

**Table 3:** Definition of YAML entry fields for material properties. This YAML file contains detailed information about the nine rock properties described in Table 1 for each rock formation. Included are the data source, units, the description of the data value, and other metadata.

Field	Sub-Field	Data Type	Description	Additional Note
source	—	string	Reference to the data’s origin	The entry here corresponds with the BibTeX citation key found in source.bib in the SDH
type	—	string	Type of the value	It can be either scalar, dictionary, or expression
value	—	float or dictionary or string	Best estimate value	If the value type is a dictionary, it is given as key/value pairs. The key refers to the “variable”.If you want to enter an expression, format it as a string, for example, 5*x+7
value_min	—	float	Minimum value	
value_max	—	float	Maximum value	
value_std	—	float	Standard deviation	
probability _distribution	sample_size	int	Number of samples	
probability _distribution	sampled_data	string or numpy list or numpy array	A scipy.stats.rv_continuous().rvs() function that generate random variates or a list of custom-generated samples	e.g.,scipy.stats.norm(loc=0,scale=1).rvs (size=100,random_state=2)
unit_str	—	string	SI unit of the property	e.g., “kg/m^3” for density
unit_base	—	list of integers	The exponent of SI base units:[kg, m, s, K, A, mol, cd]	e.g., [1, -3, 0, 0, 0, 0,0] for density
variable_name	—	string	The key or function argument in the dictionary or expression	
variable_unit _str	—	string	SI unit of the variable	
variable_unit _base	—	list of integers	The exponent of SI base units: [kg, m, s, K, A, mol, cd]	
description	—	string	Detailed lithological description on the data	
tag	agency	list of strings	A list of involved agencies	e.g., [BGR]
tag	location	list of strings	Locations for data’s origins	e.g., [Gorleben, Lower-Saxony, Germany]
tag	simplified_lithology	list of strings	A list of simplied lithologies	
tag	id	string	Unique id	UUID format with 36 characters^95^

#### Rock Property Metadata Convention

The simplified lithology field in Tables 2 and 3 identifies the rock types constituting each rock formation. Clear rock classification with unambiguous names is needed to support the data merging process. The International Union of Geological Sciences Commission for Geoscience Information Geoscience Terminology Working Group (GTWG) has been developing the internationally accepted geoscience vocabularies. These vocabularies include lithological rock names, hierarchically organized with up to six levels^72^. For simplicity, we categorized all sedimentary rocks mainly by grain size, regardless of sedimentary material type. Please note that we do not further classify limestone, mudstone, and sandstone. For example, claystone and silicate mudstone are classified as “Mudstone”. For igneous rocks, we apply a two-group classification: Crystalline igneous rocks and Fragmental igneous rocks, although the latter is not relevant for nuclear waste disposal. This classification approach is based on the Rock Classification Scheme by the British Geological Survey^73^. All fragmental igneous rocks defined in GTWG are classified as “Fragmental_Rock”, while all other groups of igneous rocks are classified as “Crystalline”. Lastly, non-carbonate salt rocks, mainly consisting of evaporite minerals, are classified as “Rocksalt”. Please note that this simplification strategy aims to assist data integration during uncertainty analysis and to provide sensible defaults when data is unavailable.

#### Geological Background

Data collection revealed that geological formations spanning political borders enable the use of international data relevant to German geological conditions. The following geological background provides the geological basis for cross-border formations and establishes guidelines for incorporating international data where German data remains insufficient.

1. Rock salt: For Mesozoic and Palaeozoic rock salt, European deposits are predominantly characterized by extensive stratiform layers and complex salt-tectonic structures formed during the Zechstein age^74^. According to BGE (2020)^75^, the most substantial rock salt formations are located within the Northern German Zechstein Basin, part of the Mid-European Permian Basin extending from eastern Britain to central Poland^76,77^. Additionally, noteworthy Mesozoic and Palaeozoic rock salt deposits are present in the Pripyat Basin in Belarus and Ukraine, the Lublin Trough in eastern Poland, and the Cheshire and Wessex Basins in southern England^78–81^. Furthermore, regarding Triassic rock salt, despite limited studies on Triassic rock salt for nuclear waste disposal in Germany, extensive research on geothermal energy, energy storage^79,80^, and large-scale geophysical surveys^82^ of Triassic rock salt offers valuable insights into its geometry and geomechanics.
2. Claystone: Three major European depositional phases, Jurassic, Triassic, and Cenozoic, are relevant due to their thick, well-compacted claystone formations suitable for repository development.
  Prevalent in Jurassic claystone, spread throughout Lower Saxony, Southern Germany, and in Southwestern Europe, including the Paris Basin and the Massif Central, is Opalinus Clay^83,84^. The Opalinus Clay formation, extensively studied by the Swiss agency NAGRA, continues from the Alpine region into Southern Germany, including the Swabian Alb and the Molasse Basin, reinforcing their importance as reference sites. Other comparable Jurassic clay-rich deposits across Europe include the Callovo-Oxfordian claystone in the Paris Basin and the Toarcian claystone in France’s Massif Central^84,85^.
  The Triassic claystone in Germany exists primarily within the Keuper rock formation^37,43^, the uppermost unit of the German Triassic, which is widespread across the German Basin. This basin extends from England, through northern Germany and Poland, stretching into southwestern Germany, Switzerland and parts of France^86^. Comparable deposits mainly occur in the western Molasse Basin, such as the anhydrite-bearing Keuper formations in northern Switzerland ^87^, and the North German Basin^43^.
  Cenozoic Claystones occur in Northern Germany and distinct regions such as the Southern German Molasse Basin and the Upper Rhine Graben^50^. The Boom Clay Formation in Belgium also represents a noteworthy Cenozoic Claystone formation. However, reduced compaction levels and relatively constrained thicknesses of up to 100 meters imply that data derived from these formations, including from the High Activity Disposal Experimental Site (HADES) underground lab, must be carefully evaluated for applicability to geological contexts in Germany^88,89^.
3. Crystalline Rock: While crystalline rock is a suitable host rock for high-level radioactive waste repositories, its utilization potential in Germany remains limited due to scarcity in extent and distribution^75^. While occurring throughout the Central German Uplands and subsided zones of the Central German Upland Range, particular emphasis must be placed on Crystalline host rocks in the Saxothuringian Zone, as it contains the Mid-German Crystalline Zone, notably hosting several large granitoid bodies^14^. The Mid-German Crystalline Zone represents a segment placed within the broader European Crystalline Zone, a geological feature stretching from the Iberian Peninsula through the Atlantic margins of Belgium and Northern France, to Central Poland and the Czech Republic^90,91^. A distinct lack of underground research facilities and data for the Saxothuringian region forces reliance on international research, such as Äspö in Sweden, Olkiluoto in Finland, Grimsel in Switzerland, and Bukov in the Czech Republic^92^. As only the Bukov underground research laboratory was situated in metamorphic rocks formed by the same Variscan orogeny that also influenced the development of relevant German crystalline bodies, well-characterized research sites comparable to crystalline rocks in Germany are strictly limited^93,94^.

### Data Processing

The inherent efficiency of the SDH derives from three core processing components: tagging, merging, and the leverage of sensible defaults. These components enable efficient data compilation for specific simulations while maintaining uncertainty quantification. The default data implementation ensures data integrity. Each of these components is further described in subsequent sections.

#### Metadata Management

Metadata management, achieved through tagging collected data, is key to tailoring specific simulation scenarios and to facilitating the reproducibility of simulations. As shown in Table 3, four tag types are added to the data: agency, location, simplified lithology, and ID. The first three tags are manually added and are defined as follows:

1. The word “agency” derives from the German words for Ministry, Society, and Office (or Ministerium, Gesellschaft, and Amt in German). If the involved party differs from the just-mentioned definition, then it is classified with the agency tag “Others”. Entities falling under the “Others” tag include the following:
  Research not done through a federal agency, e.g., universities.
  A private company or institute hired through an agency to conduct research.
2. The “location” tag refers to the data’s origin, beginning with the country and then followed by the sub-state or exact location, e.g., a city or region.
3. The previously described rock naming strategy serves as a guideline for the simplified lithology tag.

Lastly, each data point has its unique ID. It is an auto-generated UUID, namely Universally Unique IDentifier^95^, based on the contents of all other entry fields. The unique ID, automatically added as a tag, enables tracking of data used in a specific simulation, further enhancing reproducibility.

#### Merging

With a large amount of data existing, it is not surprising that different sources provide a multitude of values for a particular property. Accounting for the multitude of values while also ensuring ease of use is difficult, especially when factors such as data inconsistency, limited availability of metainformation, and uncertainties must be considered. As for the merging process, the SDH user may choose between two options. Firstly, manually choose the tagged value for a particular property from a certain source. Secondly, merge the data employing a simple methodology, then use the output for further analysis. Due to the low porosity of dense rocks, no distinction is made between values of the same type of property when merging them, as defined in Table 1. For example, both effective porosity and total porosity are treated the same when merging. When applying the simple merging methodology, two types of information are considered for storing:

1. The respective minimum and maximum values from the given data.
2. Probability distribution of an existing dataset.

To obtain distribution information, the idea is to generate a truncated normal distribution from the merged datasets to be physically meaningful. While automated merging considers all provided sources to minimize subjectivity of the analysis, it is sometimes necessary to consider expert knowledge during data preparation. In these cases, users may opt to utilize their preferred merging method by exporting the dataset prior to applying the provided merging solution. The provided merging method consists of the following:

1. If a probability distribution is not provided, create a Probability Density Function (PDF) for each data set. The recommended PDF for each entry case is listed in Table 4. Entries not qualified are excluded from the analysis. For properties with a single value and/or a maximum range, a lognormal distribution is recommended. The standard deviation is then calculated using a recommended coefficient of variation (CV), defined as the ratio of the standard deviation to the mean. Property-specific CVs are derived from the SDH data and can be obtained from the SDH source code.

**Table 4:** Recommended PDFs based on data availability. “value” denotes the best-estimate value; “value_min” and “value_max” represent the observed range; “value_std” refers to standard deviation. The “x” marker indicates a non-empty entry where data is available and recorded from the cited source. Four types of recommended distributions are provided by the SDH: 1) truncated normal distribution: Applicable if the data source provides at least the best-estimated value, minimum value, and standard deviation. 2) PERT distribution: When the best-estimated value, minimum value, and/or maximum value are available. 3) lognormal distribution: Requires at least the best-estimate value. 4) Uniform distribution: When both minimum and maximum values are available.

value	value_min	value_max	value_std	Recommended PDF	Additional Note
x	x	x	x	truncated normal distribution	
x	x	x		PERT distribution	Project Evaluation and Review Technique (PERT)^98^
x		x	x	lognormal distribution	
x	x		x	lognormal distribution or truncated normal distribution	Assume a truncated normal distribution for porosity with a maximum value of one
x	x			lognormal distribution or PERT distribution	Assume a PERT distribution for porosity with a maximum value of one
x		x		lognormal distribution	Standard deviation assumed and calculated from the property-specific CV provided by the SDH
x			x	lognormal distribution	Ensure all values are greater than zero
	x	x		uniform distribution	
x				lognormal distribution	Standard deviation assumed and calculated from the property-specific CV provided by the SDH
2. Generate samples from each PDF and keep only those within three standard deviations of the mean. Users can determine the number of samples to generate by entering the sample size, as described in Table 3.
3. Combine all sample sets and then fit them into a truncated normal distribution by calculating the mean, standard deviation, minimum, and maximum from the combined sample set.
4. Store the probability distribution, mean, standard deviation, and maximum and minimum values from the sample dataset.

#### Sensible Defaults

**Fig. 3:**
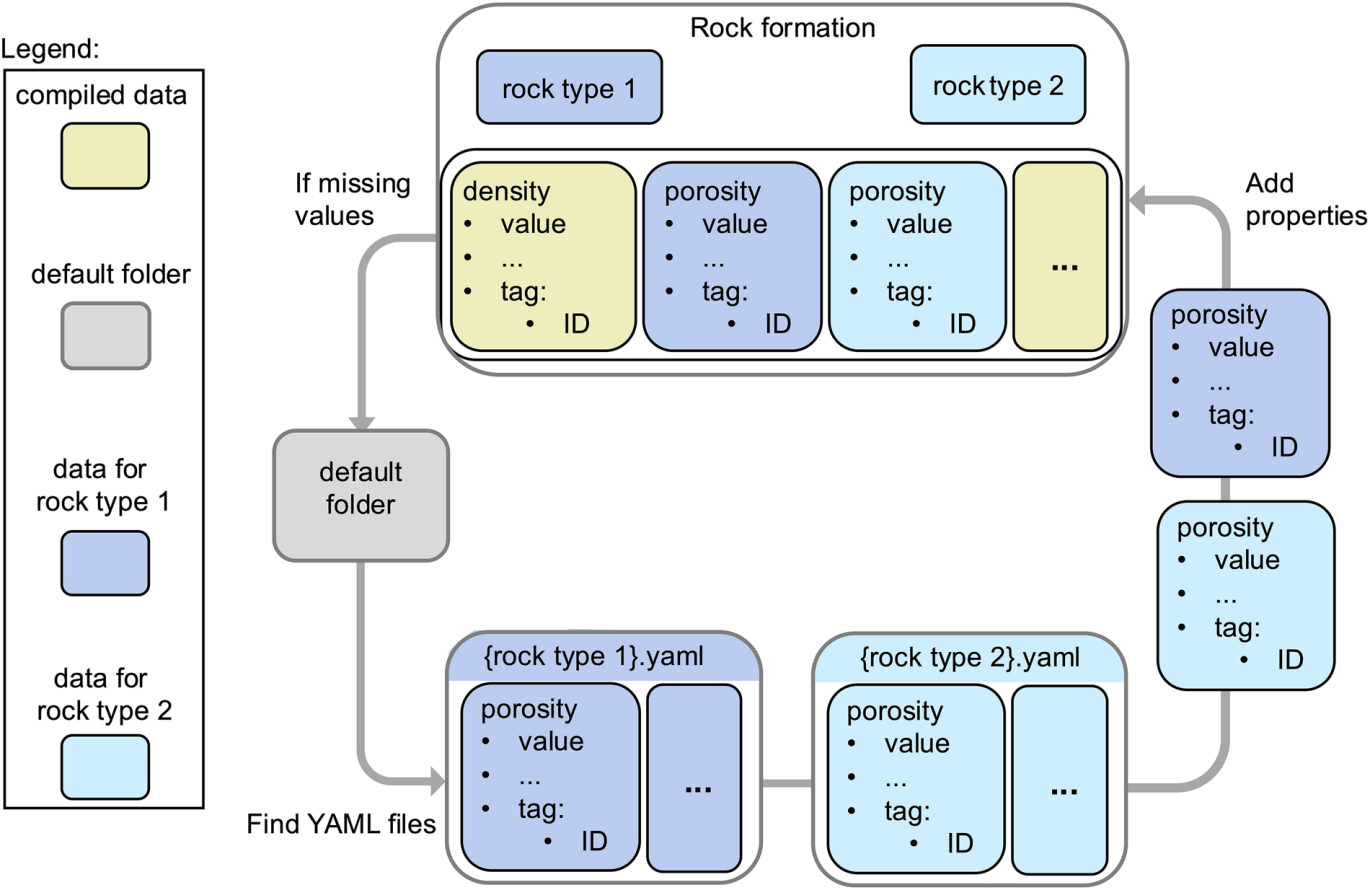
Default mechanism: An example of complementing missing values. Original data records from literature sources are colored dark yellow. The colored grey is the default folder. Default data from rock type 1 is colored dark blue, and the default data from rock type 2 is colored light blue. In this example, the missing porosity value is complemented with default values based on the rock types in the rock formation, namely rock type 1 and rock type 2.

Gaps in the data compilations naturally arise, as it would be prohibitive to restrict the SDH to those datasets that are complete. Such gaps are filled using a sensible default strategy, providing default data linked to the rock types whenever values are otherwise missing. We provide default data for the rock properties described in Table 1 and applicable to the following rock types: Conglomerate, Mudstone, Sandstone, Limestone, Dolomite, Rocksalt, and Crystalline. The default data for each rock type is calculated using the merging method on the existing dataset data. Encountering a scenario where default values become relevant, the user may choose between three options. Firstly, achieve completion by automatically filling in default values wherever values are missing. Secondly, provide data manually and tag them accordingly. Lastly, export an incomplete dataset. In the first case, if a rock property is missing for a specific rock formation, it will be retrieved from reference files named according to the rock types of that formation. Figure 3 further explains the process.

## Data Records

**Fig. 4:**
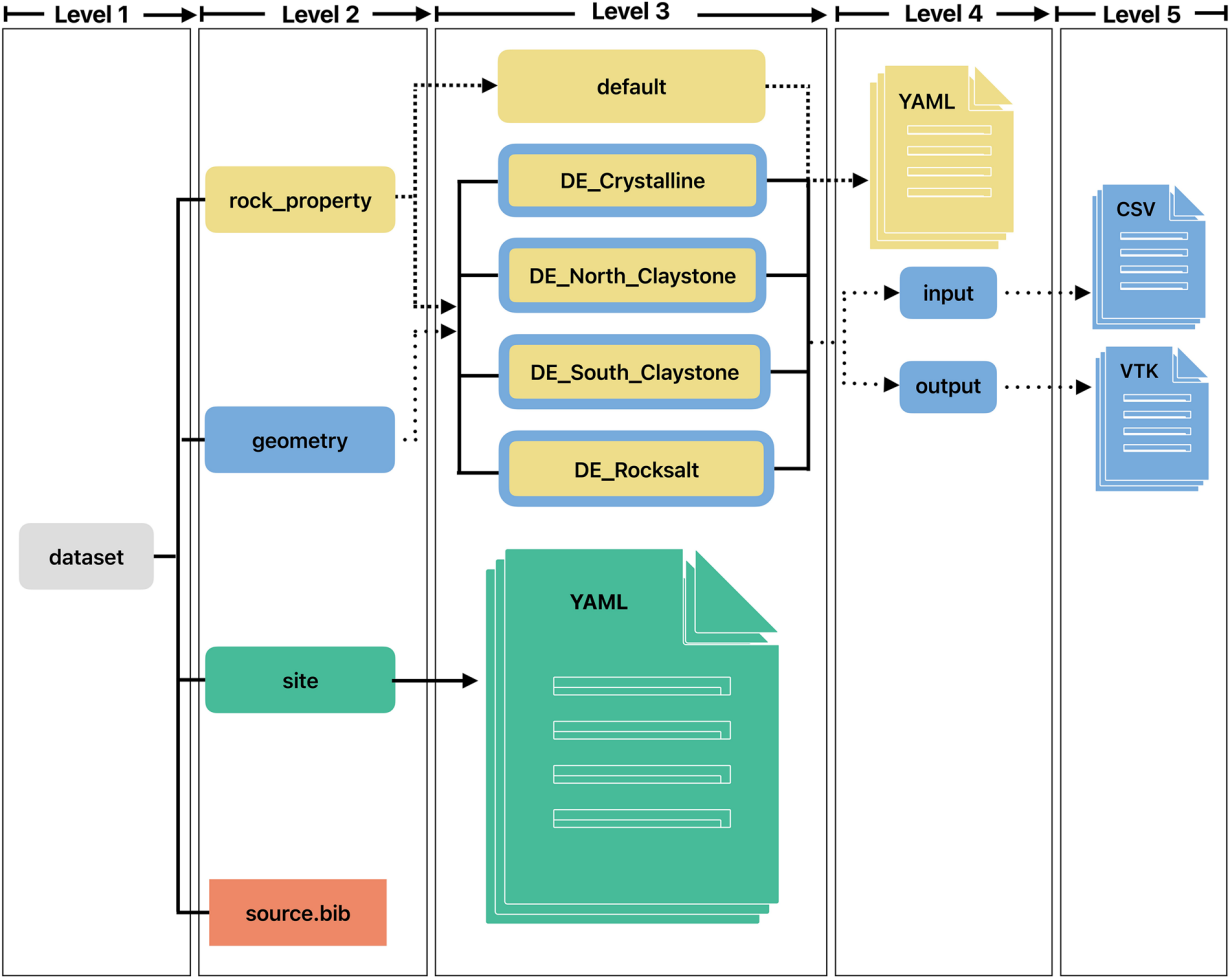
Dataset folder structure. Colors differentiate folders and subfolders associated with different parent folders. Additionally, dashed lines indicate subfolders or files in the rock property folder, and dotted lines indicate them in the geometry folder. The solid line at level 3 indicates folders existing within both parent directories. For clarity, the README files are not shown.

The static version of the SDH dataset is available in its newest version on Zenodo^96^. It consists of three main folders, which are organized hierarchically, as shown in Fig. 4. The three folders are described below:

1. site: site-specific geological information on each lithological rock formation. It refers to a potential repository capable of containing high-level radioactive waste. Each site contains data representing a generic candidate site in Germany, including a summary of its rock formations.
2. geometry: structural information on each lithological rock. Its subfolder is further divided into two folders. The first folder contains CSV files, and the second folder contains VTK files. These CSV files are used as input to create the 3D geologic structural model, and the VTK files are output for each rock formation.
3. rock property: properties for each rock formation, including nine properties described in the section on rock properties. Contained within are the YAML files for each rock formation and its default data. The default folder contains the following YAML files: Conglomerate, Limestone, Mudstone, Sandstone, Dolomite, Rocksalt, and Crystalline.

**Fig. 5:**
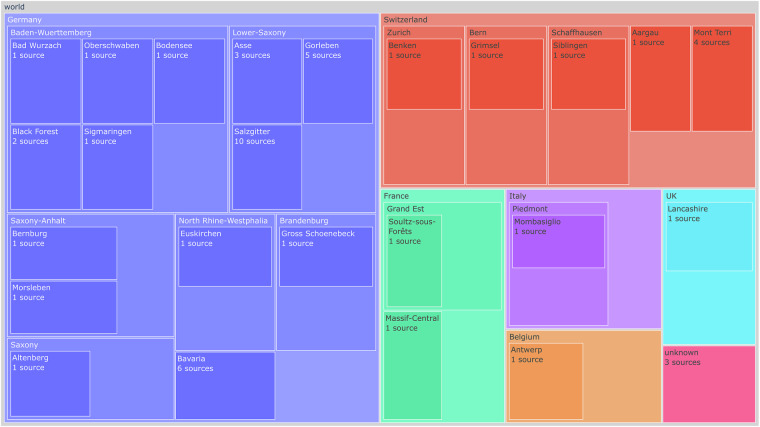
Treemap visualizing the number of sources by location. Organized in a hierarchical structure, the treemap begins with the world, then specifies individual countries, and is followed by a sub-state or, if known, an exact location, e.g., a town or municipality. Different colours indicate different countries, while colour intensity indicates the precision of the location. The figure is generated using Plotly^101^.

The data comprises 50 literature sources^14–63^, including peer-reviewed articles and official government reports. The source for each data value described in Table 2 and 3 is linked to a BibTeX citation key. All sources, with their corresponding BibTeX entries, are listed in the source.bib file. Figure 5 shows the distribution of data sources by location.

## Technical Validation

The Smart Data Hub is the tool to be used as a starting point to facilitate simulations, as it combines the key principles of reproducibility, accessibility, and transparency, all of which are essential to finding a final repository location. Ensuring the FAIR principles, the SDH serves as a strong foundation to build upon, especially when the stakes are high. Under these circumstances, informed decisions require reliable data records and the SDH outputs.

The SDH sourced data from governmental reports or peer-reviewed articles, guaranteeing its reliability. Detailed metainformation for each dataset provides the source, including the agency involved, the location, and a detailed lithological description, making everything transparent. A unique ID is provided to each source, further improving traceability. As laid out in the Methods section, tagging, merging, and sensible defaults are leveraged with a scripted programming language, making the processes involved more transparent and less prone to oversight, hence increasing overall accuracy.

The SDH systematically addresses the four focal points established at the beginning of the paper through its three core capabilities: tagging, merging, and sensible defaults. Working either independently or in combination, they enable 1) comprehensive data acquisition, followed by 2) intelligent compilation methods supporting various data configurations associated with a certain model setup, 3) explicit numerical input data uncertainty quantification and integration; and last, 4) strict enforcement of transparency and reproducibility standards.

Output from the SDH includes a 3D geological model with detailed rock property annotations, which greatly benefits from its three key capabilities. Please note that the models provided in the SDH are default geological models. However, users have the flexibility to use their own models while simultaneously accessing data from the dataset. The annotated rock properties are stored in YAML files with an example demonstrating how these capabilities work together in Tables 5 and 6.

**Table 5:** Annotated P-wave velocity merged from data tagged with “BGR” for the Granite formation. It includes details on the proposed PDF, metadata about the original datasets, and an ID for this merged dataset.

Field	Sub-Field	Entry
source	—	merged
type	—	scalar
value	—	5340.93
value_min	—	3125.44
value_max	—	7276.21
value_std	—	400.65
probability_distribution	sample_size	1000000
probability_distribution	sampled_data	scipy.stats.truncnorm(-5.53, 4.83, loc=5340.93, scale=400.65).rvs(size=1000000, random_state=21)
unit_str	—	m/s
unit_base	—	[0, 1, -1, 0, 0, 0, 0]
description	—	A truncated normal distribution fitted from 4 combined datasets with ids:7cd75673-f1cc-5219-bebc-d2a80a717a91, dbd050a8-58eb-5a70-b1e0-dcd26e68b295, 9133dc67-9ea4-5ea4-bfd6-62ea62d05014, e31c19ee-c7cb-5959-92f4-d31aa9be3206
tag	ID	ca63bdd4-01ca-5279-8bcc-c0e14f6ca71b

The mean, standard deviation, maximum, and minimum values, as well as the probability distributions in Tables 5 and Fig. 6, were calculated using the data merging method. Table 5 shows the results of four merged datasets containing P-wave velocity measurements tagged with “BGR” for the Granite formation in the crystalline host rock area.

**Table 6:** Loaded S-wave velocity from the default folder for the Granite formation tagged with “BGR”. It includes details on the proposed PDF, metadata about the original datasets, and an ID for this default dataset.

Field	Sub-Field	Entry
source	—	default
type	—	scalar
value	—	2158.62
value_min	—	1178.04
value_max	—	3434.84
value_std	—	356.23
probability_distribution	sample_size	1000000
probability_distribution	sampled_data	scipy.stats.truncnorm(-2.75, 3.58, loc=2158.62, scale=356.23).rvs(size=1000000, random_state=21)
unit_str	—	m/s
unit_base	—	[0, 1, -1, 0, 0, 0, 0]
description	—	A truncated normal distribution fitted from 3 combined datasets with ids: 14eb4543-2d4e-5832-b004-095bb45c79b4, fa363cab-9c14-58c2-b20d-6ea412f6fe48, 8f3a9174-81b5-5a5e-929f-7961f9127bfc
tag	ID	d3ba3320-f314-5145-ac4e-9f7ecde2a636

In this specific scenario, missing rock properties, including intrinsic permeability, specific heat capacity, electrical resistivity, and S-wave velocity, are sourced from the sensible defaults. Table 6 presents the S-wave velocity values loaded from the default folder.

Overall, the application of these three capabilities yields high levels of reproducibility, accessibility, and transparency. It therefore fulfils the four focal points established in the introduction: comprehensive data acquisition, customization of specific simulation scenarios, and data integrity. Furthermore, being the fourth focal point, the generated data facilitates subsequent modelling and uncertainty quantification, ultimately supporting decision-making in the nuclear waste site selection process.

## Data Availability

The dataset associated with this article is version v1 and is published on Zenodo (10.5281/zenodo.19886769) under a CC BY 4.0 license.

## Code availability

The code for processing the data, including all methods described in this paper, is available on Zenodo (10.5281/zenodo.19922612)^97^ and GitHub at the following link: https://github.com/mbd-rwth/smart_data_hub.git. This article corresponds to version v1.0.2.
